# From Green to Brown: Characterization of the Fast Alteration of Modern Greenish Enamels in Glass Windows

**DOI:** 10.1002/cplu.202500205

**Published:** 2025-08-07

**Authors:** Teresa Palomar, David G. Calatayud, Mohamed Oujja, Laura Maestro‐Guijarro, Mario Aparicio, Jadra Mosa, Bettina Koppermann, Léonie Seliger

**Affiliations:** ^1^ Instituto de Cerámica y Vidrio (ICV) CSIC c/Kelsen 5. Campus de Cantoblanco 28049 Madrid Spain; ^2^ Unidade de Investigação VICARTE—Vidro e Cerâmica para as Artes NOVA School of Science and Technology Universidade Nova de Lisboa Hangar III—Campus da Caparica 2829–516 Caparica Portugal; ^3^ Departamento de Química Inorgánica Universidad Autónoma de Madrid Facultad de Ciencias c/ Tomas y Valiente 7 28049 Madrid Spain; ^4^ Instituto de Química Física Blas Cabrera (IQF) CSIC c/ Serrano, 119 28006 Madrid Spain; ^5^ Canterbury Cathedral 8A The Precincts Canterbury Kent CT1 2EG UK

**Keywords:** alterations, glasses, green enamels, leads, stained‐glass windows

## Abstract

In 2009–2014, new glass panels were produced by the Stained‐Glass Studio of Canterbury Cathedral (UK) and installed in St Peter's Church (Little Barrington, Burford), St Lawrence's Church (Mereworth, Kent), and Canterbury Cathedral. After a few years, some spots and stains have appeared on the greenish areas of the panels. The common factor is that the panels were produced using the same green enamel. The present work reports on the studies of the observed alterations in green enamels to propose possible degradation mechanisms. These studies are based on using different analytical techniques such as optical microscopy, scanning electron microscopy energy dispersive X‐ray spectroscopy, inductively coupled plasma mass spectrometry, laser‐induced breakdown spectroscopy, X‐ray diffraction, *µ*‐Raman spectroscopy, profilometry, X‐ray photoelectron spectroscopy, and UV–vis spectroscopy. The characterization of the fragments from the original panels showed that a brown layer of dark lead compounds was formed on the green enamel. The alteration occurred in a 3‐step alteration mechanism divided into Pb^2+^ lixiviation from the enamel, formation of hydrocerussite (2PbCO_3_·Pb(OH)_2_), and transformation into scrutinyite (*α*‐PbO_2_) and plattnerite (*β*‐PbO_2_).

## Graphical abstract

Spots and stains appeared on the modern green enamel from different stained‐glass windows as a consequence of environmental alteration. The multianalytical characterization showed a 3‐step alteration mechanism divided into Pb^2+^ lixiviation from the enamel, formation of hydrocerussite (2PbCO_3_·Pb(OH)_2_), and transformation into scrutinyite (*α*‐PbO_2_) and plattnerite (*β*‐PbO_2_).

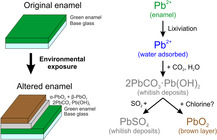

## 1 Introduction

Stained‐glass windows have been commonly painted with enamels since the 15th century.^[^ ^1^ ^]^ They are layers of colored glass that melt at lower temperatures than the substrate glass onto which they are applied.^[^ ^2^, ^3^ ^–^ ^4^ ^]^ Enamels are different from grisailles, which are another glass‐based paint formed by a mixture of a lead‐rich glass with grounded metal oxides, and it is commonly applied for drawing contours, adding shadows, and textures to figures.^[^ ^5^ ^]^ Both painting materials can be very influenced by the environment, especially if applied on an outdoor surface.

One of the most common causes of alteration is the breakage of glass due to vandalism or frame movements. Regarding chemical degradation, the environmental alteration of historical glasses has been thoroughly evaluated since the 1990s, focusing on the corrosion phenomena, and the improvement of their conservation measures.^[^ ^6^, ^7^, ^8^ ^–^ ^9^ ^]^ However, the number of studies regarding glass painting degradation is very scarce thus far. Regardless of their nature, the most common cause of alteration of these paintings is the environmental humidity (rain, condensation, etc.), which creeps in through microfissures, thus inducing the progressive powdering of the painting, the dealkalinisation of the substrate glass, and the loss of adherence of the paint layers.^[^ ^10^ ^]^ The alteration degree depends on their chemical composition, their thickness, their degree of vitrification, and the presence of bubbles,^[^ ^2^ ^,^ ^10^ ^]^ which are directly related to the production technology. In addition to humidity, enamels are extremely sensitive to thermal shock, which can induce the formation of fissures and detachments due to the incompatibility of enamels with the glass support.^[^ ^11^, ^12^, ^13^, ^14^ ^–^ ^15^ ^]^ Even blue enamels can experience a change in their color due to their reaction with distilled water.^[^ ^16^ ^]^

The Stained‐Glass Studio of Canterbury Cathedral (UK) is dedicated to the conservation, restoration, and protection of stained glass from the 12th to the 20th Century. The studio also designs and creates new stained‐glass windows for ecclesiastical buildings, historic buildings, and private homes throughout the UK.^[^ ^17^ ^]^

**Figure 1:**
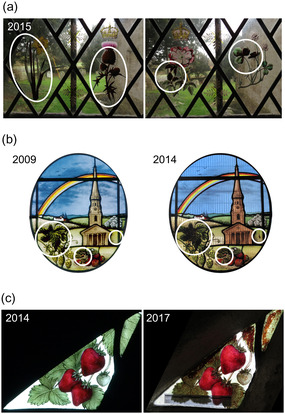
Examples of the brownish spots and stains on the greenish areas of the panels after a few years of their installation. a) St Peter's Church in Little Barrington, Burford. b) St Lawrence's Church in Mereworth, Kent. c) Canterbury Cathedral, Willet window.

A few years ago, the Studio observed that some glass panels produced by them and installed in St Peter's Church (Little Barrington, Burford), St Lawrence's Church (Mereworth, Kent), and Canterbury Cathedral experienced the formation of brownish spots and stains on the greenish areas of the panels (**Figure** **1**a–c), which faced the outside at all three locations. In all of them, a green transparent enamel supplied in the 1980s/90s was used (Glassmarket 11,934). Fortunately, this enamel is no longer available.

St Peter's Church in Little Barrington, Burford, is a well‐ventilated church. Stained glass containing enameled pieces was installed in the east‐facing window of the side aisle at ca 2.5 m above ground. There are no new furnishings that might be off‐gassing. Candles may be burned in the church on special occasions, but in small numbers, not contributing significantly to air pollution. Due to its location against the protruding chancel and surrounding trees, the window is unlikely to get any significant amounts of direct sunshine, so diurnal temperature changes are likely to be gradual and moderate.

St Lawrence's Church, Mereworth, has a large, well‐ventilated interior without new furnishings and no frequent burning of candles. This window faces south, there are no trees immediately close to it, and the glass will experience significant diurnal temperature changes, ranging from 5 °C to well over 40 °C on a sunny winter's day. The effect of browning over five years was not so pronounced, but in one spot, very noticeable.

Both in Little Barrington and in Mereworth, the green enamel is applied to the exterior face of the glass to add color to certain areas. In addition, brown vitreous grisaille paint is applied to the interior face to produce outlines and shading. The two different forms of applied decoration (enamel and grisaille) are therefore not in contact with each other.

In the third location, the enamels from the windows in the cloisters of Canterbury Cathedral showed very rapid and severe deterioration. There, it faces west and receives direct sunshine only in the late afternoon. The cloisters are not fully glazed; only the tracery parts of the arcades have glazing, the main lancets are always open to the elements, so there are almost no differences in environmental conditions between the interior and the exterior of the building.

In the Canterbury window, the painter applied both enamel and grisaille paint to the interior glass surface. The grisaille paint was applied first and kiln‐fired (fused) to the glass. The green enamel was applied afterward and fired at a slightly lower temperature in a second kilning process.

The only common denominator between the three locations (other than the use of the same enamel) is that they are all in a rural environment. They do not share orientation or diurnal temperature changes. No significant sources of air pollution are known to exist in any of these locations.

The present study focuses on the characterization of the original enamels and a set of altered‐enamel samples, as well as on the identification of the degradation mechanisms leading to the formation of brown layers. This research will be especially useful for artists and conservators who work with glassy enamels, mainly with this specific enamel.

## 2 Experimental Section

### 2.1 Samples

**Figure 2:**
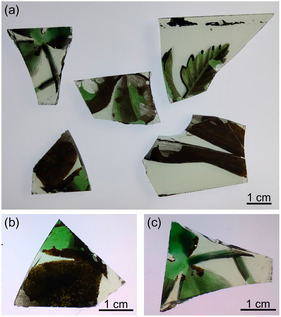
a) Set of samples observed in transmitted light from St Peter's church in Little Barrington, Burford. b,c) Two samples showing the brown degradation layer.

A set of original samples from St Peter's Church in Little Barrington, Burford, has been characterized (**Figure** **2**). Because in this location the green enamel was applied to one glass surface and the grisaille paint to the other, the brown alteration layer was clearly not a deliberately applied grisaille paint. Moreover, samples taken of the brown alteration layer could not inadvertently be contaminated by grisaille paint.

### 2.2 Analytical Methods

The glass samples with green enamel and brown layers have been characterized by the following techniques: optical microscopy (OM), field emission scanning electron microscopy with energy dispersive X‐ray spectroscopy (FE‐SEM‐EDS), profilometry, ultraviolet–visible spectroscopy (UV–VIS), laser‐induced breakdown spectroscopy (LIBS), confocal Raman microscopy, X‐ray photoelectron spectroscopy (XPS) and, grazing incidence X‐ray diffraction (GIXRD).

OM observations were carried out with a reflected light microscope Nikon SMZ1000 coupled to an Axiocam 105 color controlled with the software Zen2012 (blue edition) from Carl Zeiss Microscopy GmbH, 2011.

Morphological analysis of the glass surfaces was performed by field emission scanning electron microscopy (FE‐SEM) HITACHI S‐4700. For elemental chemical analysis by energy dispersive X‐ray spectroscopy (EDS) a NORAN system six was connected to the FE‐SEM.

The samples surfaces were observed in a Zeta Instruments optical profilometer model Zeta‐20 used as an optical microscope.

UV–vis spectroscopy analysis was carried out using the Analytik‐Jena Specord 200 Plus spectrophotometer equipped with deuterium (UV, 190–318 nm) and tungsten (UV–visible, 318–1100 nm) lamps. A slow scan of 250–900 nm was carried out and the samples were placed inside an integrating sphere for diffuse reflectance of 120 mm.

LIBS analysis was performed at ambient atmospheric conditions, at the surface and in‐depth (stratigraphic study) of the glass samples using laser excitation at 266 nm (fourth harmonic of a Q‐switched Nd: YAG laser (Lotis TII, LS‐2147), 15 ns pulses, 1 Hz repetition rate. LIBS spectra were recorded using a 0.2 m Czerny–Turner spectrograph (Andor, Shamrock Kymera‐193i‐A) equipped with a grating of 1800 grooves/mm (blazed at 265 nm) and coupled to a time‐gated intensified charge‐coupled device (ICCD) camera (Andor Technology, iStar CCD 334, 1024 × 1024 active pixels, 13 μm × 13 μm pixel). The ICCD detector was synchronized with the Q‐Switch output electrical signal that triggers the laser pulse. The laser beam was directed to the surface of the samples by the use of mirrors at an incidence angle of 45°. Focusing with a 10 cm focal length lens allowed fluences in the range of 5–9 J cm^−2^ to be achieved. The shot‐to‐shot laser energy fluctuation was less than 10%. LIBS spectra were recorded in the 240–600 nm wavelength range using a step and glow mode at intervals of 30 nm. For the stratigraphic analysis, the LIBS spectra were recorded by applying several laser pulses in the same area of the samples at 30 nm wavelength intervals centered at 285, 325, 360, 400 and 500 nm. The spectra were recorded at a 0.17 nm resolution with a gate delay and width of 200 ns and 3 μs, respectively. For higher wavelengths, a cut‐off filter at 300 nm was placed in front of the entrance window of the spectrograph to reduce the scattered laser light and to avoid second‐order diffractions. Each LIBS spectrum resulted from a single signal acquisition as it provides good signal/noise ratios. The stratigraphic analyses were done applying 50 laser pulses in the same point. The different thickness analyzed depended on the nature of the materials.

Raman spectra were recorded using a confocal Raman microscopy integrated with atomic force microscopy (AFM) on a CRM‐Alpha 300 RA microscope (WITec, Ulm, Germany) equipped with a Nd:YAG dye laser (maximum power output of 50 mW at 532 nm). The incident laser power was 35 mW and the Raman spectral resolution was down to 0.03 cm^−1^. The sample was mounted on a piezo‐driven scan platform with a lateral positional accuracy of 4 nm and a vertical positional accuracy of 0.5 mm. XY and XZ scan profiles were obtained using a studied area of 10 × 10 µm. The collected spectra were analyzed using Witec Control Plus software.

XPS measurements were performed on a FlexPS‐ARPES‐E spectrometer (SPECS) with Al K*α* X‐ray radiation as the X‐ray source for excitation.

Finally, GIXRD measurements were collected with a Bruker AXS D8 diffractometer equipped with a cobalt X‐ray tube. A Goebel mirror optics was applied to obtain a parallel and monochromatic X‐ray beam. A current of 30 mA and a voltage of 40 kV were employed as tube settings. Operational conditions were selected to obtain X‐ray diffractograms with sufficient counting statistics. XRD data was collected with a beam incidence angle of 1° between 20 and 100° with a step size of 0.03° and a counting time of 3 s/step. The analysis and identification of the peaks was done with the program Match! 3. The background of the spectra was subtracted and the spectra were smoothed with the Savitzky–Golay method in polynomial order 2 with the program Origin 9.

## 3 Results and Discussion

### 3.1 Morphology

**Figure 3:**
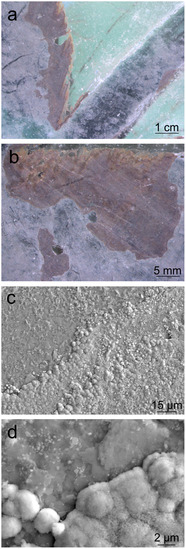
a,b) Binocular glasses images of the brown layer and the white deposits on the green enamel. c,d) Micrographs showing the limit of brown layer and white deposits.

The original samples showed a homogeneous brown layer over the surface of the green enamel and not on the colorless base glass, confirming that an alteration of the enamel was produced (**Figure** **3**a,b). This brown layer commonly appeared together with a whitish layer. The deposits appeared specifically on the enameled areas, far from the lead rods. This fact suggests discarding the hypothesis that the observed alteration comes from lead rods because they do not show any type of runoff from the metallic frame toward the enamel location (Figure 2a and 3a).

**Figure 4:**
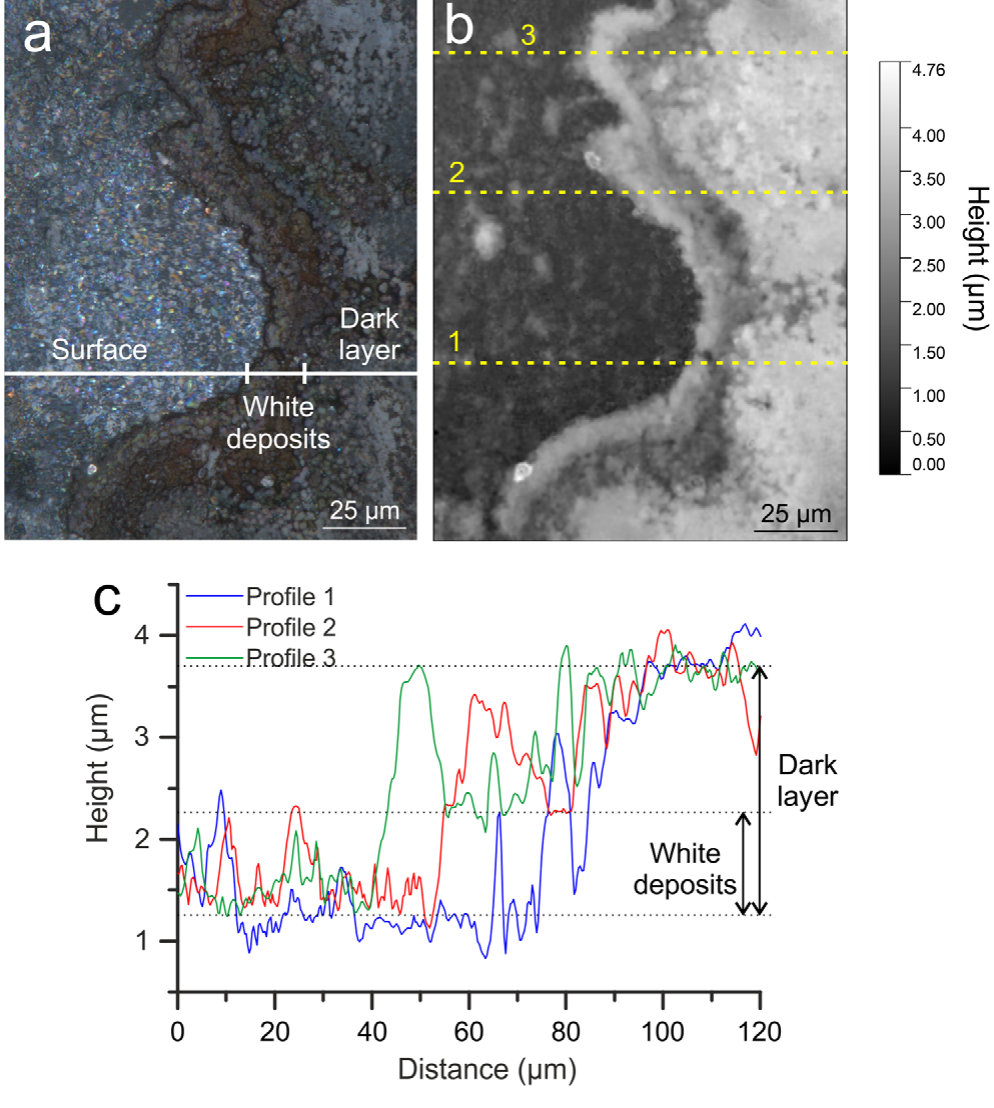
a) Profilometer image and b) profilometer mapping of the border of brown deposits over the green enamel, c) profilometer profiles extracted from the mapping.

At high magnification, it is possible to distinguish the different layers over the green enamel (Figure 3c). The brown layer appeared microscopically rough with the presence of rounded deposits, and the white one is formed by flat crystals dispersed at the surface (Figure 3d). The laser profilometer showed that the brown layer was formed on the green enamel and has a thickness of around 3 µm, and the white deposits have ≈1 µm thickness (**Figure** **4**a–c).

### 3.2 Chemical Composition

FE‐SEM‐EDS analyses showed a stable soda‐lime silicate glass and the different areas of the enamel, both the whitish and the brown layers, with a very high concentration of lead (**Table** **1**). This value of Pb, >90 wt%, could be related to the chemical composition of the enamel. However, it is observed that the brown enamel showed a slightly higher content of PbO, which could be related to the precipitates observed by FE‐SEM (Figure 3c). The analysis of the whitish layer showed the presence of Cr_2_O_3_ and a higher content of CoO, which could be the chromophores of the green enamel.

**Table 1:** FE‐SEM‐EDS analyses (wt%) of the different areas in the surface of an original glass sample.

	Na_2_O	MgO	SiO_2_	K_2_O	CaO	Cr_2_O_3_	CoO	PbO
Support glass	11.3	1.7	76.1	0.2	10.6	–	–	–
Whitish layer (over the enamel)	–	–	5.5	–	0.7	0.5	1.5	91.8
Brown layer (over the enamel)	–	–	4.1	–	0.4	–	0.9	94.7

**Figure 5:**
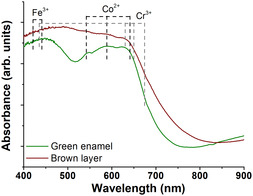
Absorbance spectra of the green enamel and brown layer on the original sample.

The visible spectrum of the green enamel confirmed the presence of cobalt (Co^2+^) and few of chromium (Cr^3+^) and iron (Fe^3+^) as chromophores (**Figure** **5**). The spectrum of the brown layer also shows the presence of the same elements; however, the bands are saturated due to the opacity of this layer.

**Figure 6:**
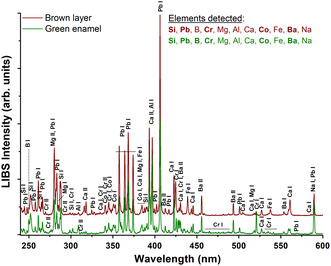
LIB spectra of the brown layer and green enamel on an original sample in the spectral range 240–600 nm. The LIB spectra were collected in step and glow mode at intervals of 30 nm with a single laser pulse. The time delay and gate width were 200 ns and 3 μs, respectively. The assignment of the line emissions is done using the NIST database.^[^ ^44^ ^]^ The main detected components are indicated in bold.

The chemical composition of the green enamel and the brown layer determined by LIBS (**Figure** **6**) agrees with the SEM‐EDS results (Table 1). Both areas showed the same major and minor elements. However, the content of boron is higher in the green enamel and the lead content is higher in the brown layer. This behavior agrees with alteration processes experienced by the enamel. Enamels commonly use boron as a former element to decrease the melting temperature;^[^ ^18^, ^19^ ^–^ ^20^ ^]^ however, this element is very susceptible to being lixiviated as a consequence of the environmental exposure.^[^ ^20^ ^]^

LIB spectra from enamel and brown layers show almost similar qualitative elemental composition. The LIB spectrum from green enamel shows peaks corresponding to cobalt and chromium emissions, related to the chromophores (Figure 5). On the other hand, the brown layer has a higher content of lead due to the formation of leaded surface deposits. The calcium is also slightly higher in this layer compared to green enamel area, and it could be a surface contamination from the environment or wall runoff, or due to the installation process (such as the use of putty).

**Figure 7:**
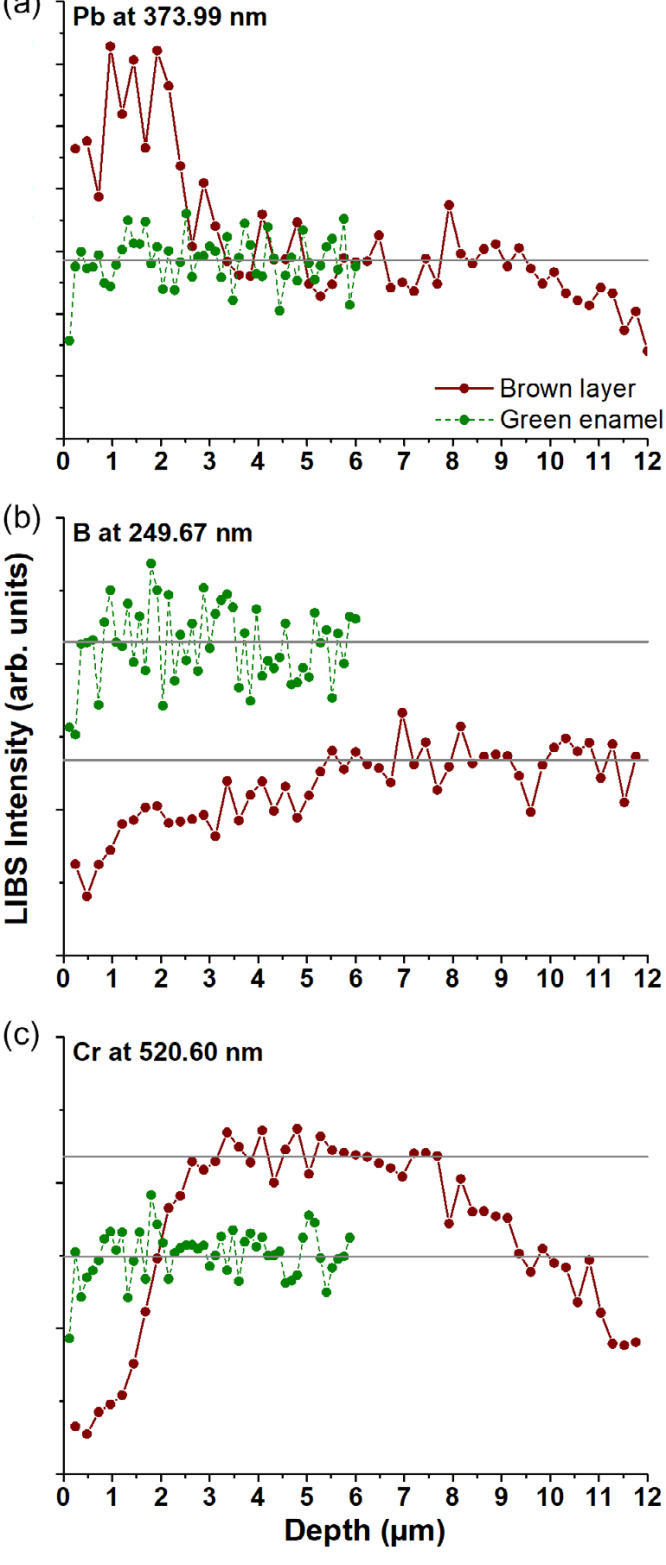
LIBS stratigraphic analysis of the original sample in the brown layer and green enamel for the elements: a) Pb at 373.99 nm, b) B at 249.73 nm and, c) Cr at 520.60 nm. The profiles correspond to 50 laser pulses.

LIBS stratigraphic analyses carried out on green enamel and brown layer show different behaviors depending on the considered element. In the case of lead in the brown layer (**Figure** **7**a), it is observed a high content at the surface and then it starts decreasing after around 3 µm before reaching a stabilized value similar to the corresponding to green enamel. This behavior agrees with the formation of a layer enriched in lead at the surface over the green enamel. In the case of the boron (Figure 7b), in the green enamel, its content is higher because boron forms part of the glassy structure, showing stable and constant values across its thickness. However, in the brown layer, the content is low during the first 3 µm, where the brown layer was formed, then a progressive increase until its stabilization at around 5 µm is observed. The boron is easily lost from the surface as a consequence of the environmental alteration,^[^ ^20^ ^]^ being its value increased up to the value of the unaltered chemical composition of the enamel. The observed differences in the stabilized values of boron could be related to different inhomogeneities in the composition across the surface of the sample. Regarding the chromium (Figure 7c), which is a chromophore of the green enamel, it shows a stable value in the enamel. However, for the brown layer, it is observed a very low content during the first 3 µm, due to the presence of the lead layer, and then started increasing till reaching a stabilized value until around 8 µm. After that, the chromium content progressively decreases, being the last values corresponding to the colorless base glass composition. The LIBS intensity depends on the area analyzed and the thickness of the surface layers. Significant differences were observed in the LIBS stratigraphic analysis of boron and chromium, because they are minor elements in the chemical composition; however, lead is a major element, and the difference in intensity is relatively lower. These results indicate that the lixiviation of lead and the low content of chromophores, previously present in the enamels composition, at the outer layers are responsible of the dark hue of the deposits on the enamel.

### 3.3 Structural Analysis

**Figure 8:**
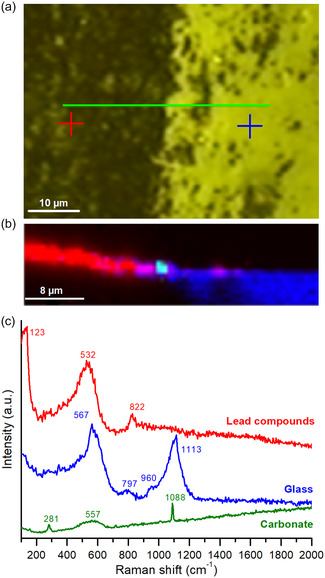
Confocal micro‐Raman analysis of the original glass: a) XY image of the analyzed surface, the green line indicates the XZ analysis, b) XZ Raman depth scan showing in false color the areas with similar spectra, c) Raman spectra identified in the analysis in depth for comparison. The colors of the plotted spectra resume the false colors used in the XZ mapping.

The different areas in the original samples were also analyzed by confocal micro‐Raman. In the linear scan of the border of the brown layer, shown in **Figure** **8**a, three different spectra were collected related to the different areas (Figure 8b,c). In blue, it is represented the spectrum with the main bands at 567 and 1113 cm^−1^. This spectrum corresponds to the composition of a common glass, such as the base glass.^[^ ^21^ ^]^ The former band at 567 cm^−1^ corresponds to the bending bonds of the Si—O bond, and the band at 1113 cm^−1^ to the stretching modes of the same Si—O bond. The spectrum from the analysis carried out on the brown layer is shown in red. An intense peak at 123 cm^−1^, a broad band at 532 cm^−1^ and a small peak at 822 cm^−1^ were detected (Figure 8c). This spectrum is consistent with the red lead (Pb_3_O_4_), where the peak at 123 cm^−1^ is the O—Pb(IV)—O bending vibration, and the band at 532 cm^−1^ is the stretching of the Pb(IV)—O bond.^[^ ^22^ ^,^ ^23^ ^]^ The Raman peak at 822 cm^−^^1^ is often associated with As‐O (arsenate) stretching modes, probably ascribed to alkaline earth arsenate, specifically the *ν*_3_ antisymmetric stretching vibration (F_2_).^[^ ^24^ ^]^ On the other hand, a possible attribution of this Raman band to Co_2_SiO_4_, which is typical for a Co^2+^‐ion (chromophore in the enamel) dissolved within a glass silicate network, is not ruled out.^[^ ^25^ ^]^

The presence of Pb_3_O_4_ was not observed by any of the other techniques and it could be formed as a degradation of PbO_2_ caused by the effect of the laser power during Raman analyses.^[^ ^23^ ^,^ ^26^ ^]^ Finally, in the area of contact between the dark layer and the glass, some deposits of calcium carbonate appeared with intense peaks at 281 (T, translational lattice mode of the group CO_3_^2−^) and 1088 cm^−1^ (*ν*1, symmetric stretching vibration of the group CO_3_^2−^) (Figure 8c).^[^ ^27^ ^,^ ^28^ ^]^

**Figure 9:**
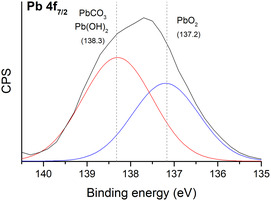
High resolution XPS spectra of the Pb 4*f*_7/2_ band in the brown layer.

The XPS analysis of the brown layer also confirmed the presence of different lead species (**Figure** **9**). The contribution of PbO_2_ and the presence of lead carbonate/hydroxide were confirmed. The formation of the carbonate and hydroxide is a common alteration produced in metallic lead,^[^ ^29^ ^]^ and it could also be produced in the lead ions (Pb^2+^) lixiviated to the surface as a consequence of the alteration of the enamel. However, the oxidation of these ions to Pb^4+^ requires high electrochemical potential and high pH.^[^ ^30^ ^]^

**Figure 10:**
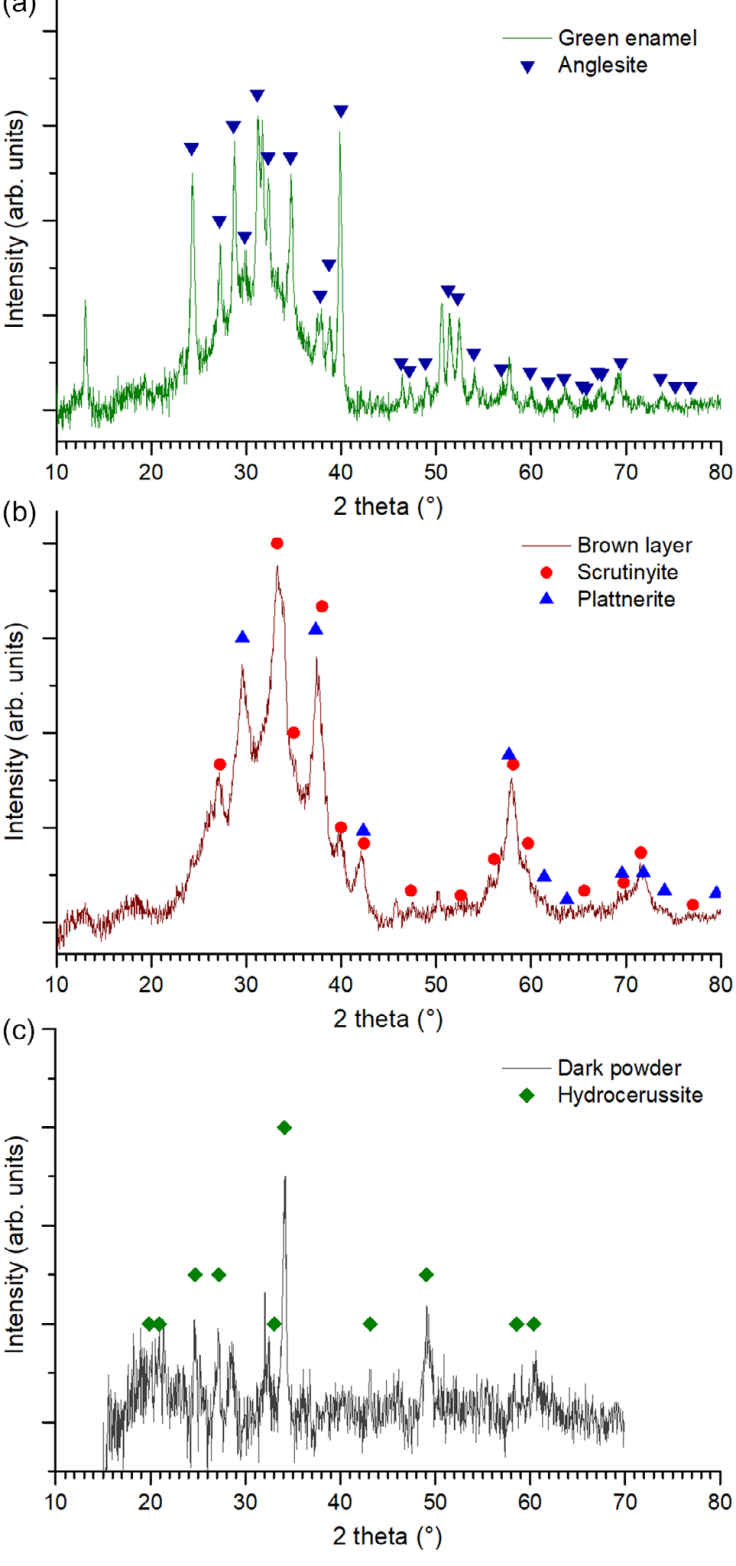
GIXRD patterns of a) green enamel, b) brown layer, and c) dark powder scratched from the surface.

GIXRD confirmed the presence of crystalline phases on the altered area. Anglesite was detected in the green enamel (PbSO_4_) (**Figure** **10**a). This compound is not common as part of the enamel composition,^[^ ^18^ ^,^ ^31^ ^]^ but it could be a product of an advanced alteration process. The formation of carbonate deposits is common in atmospheric alteration of glass; however, the sulfate species are, commonly, more stable (lower Gibbs free energy of formation) and, therefore, they are formed over long terms with carbonates as intermediate species.^[^ ^32^ ^,^ ^33^ ^]^ The detachment of the brown layer in some areas (Figure 3a,b), could leave the carbonate deposits exposed to atmospheric gases accelerating the sulfation mechanism. Dark PbS, which can be the result of the possible combination of H_2_S, present in the environment, with Pb_3_(CO_3_)_2_(OH)_2_ was not detected on the alteration layers (Figure 9 and 10), then the hypothesis that the precursor H_2_S was involved in the reaction is discarded.

In the brown layer, scrutinyite (*α*‐PbO_2_) and plattnerite (*β*‐PbO_2_) were identified (Figure 10b), both polymorphic species of the lead dioxide agreeing with the XPS analysis (Figure 9). The common alteration of metallic lead involves the formation of plumbonacrite (6PbCO_3_·3Pb(OH)_2_·PbO), hydrocerussite (2PbCO_3_·Pb(OH)_2_) and cerussite (PbCO_3_).^[^ ^29^ ^,^ ^34^ ^]^ However, the oxidation of the Pb^2+^ to Pb^4+^ is not a frequent phenomenon because this oxidation requires high electrochemical potential and high pH. Nevertheless, recently, this alteration reaction has been detected in wall paintings,^[^ ^35^, ^36^, ^37^ ^–^ ^38^ ^]^ and metallic sculptures.^[^ ^39^ ^,^ ^40^ ^]^ Most of the studies associates the alteration to the presence of a basic substrate, humidity, and sunlight, or pollutants, which are common alteration factors without proposing any specific mechanism. However, Avranovich Clerici^[^ ^36^ ^]^ suggested that the oxidation of lead white triggered by active chlorine species fosters the formation of black Pb(IV).

Finally, in the dark powder scratched from the surface (Figure 10c), hydrocerussite (2PbCO_3_·Pb(OH)_2_) was detected, which could be an intermediate species in the mechanism, as proposed in the following section.

### 3.4 Proposed Alteration Mechanism

**Figure 11:**
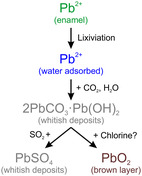
Proposed mechanism of alteration leading to the formation of white and brown layers.

According to the results of this study, the proposed alteration mechanism occurs through 3 steps, and is summarized in **Figure** **11**.

The alteration begins with the leaching of the lead ions that have formed part of the enamel structure due to the attack of the water from the environment.^[^ ^41^ ^]^ This ion dissolved in the surface water can react with the environmental gases, such as CO_2_, to form lead carbonates, such as hydrocerussite (2PbCO_3_·Pb(OH)_2_) and cerussite (PbCO_3_), which are among the most stable compounds in the PbO‐CO_2_‐H_2_O system.^[^ ^34^ ^]^ In metallic objects, these compounds commonly form a whitish, powdery and low‐adhesion alteration layer on the lead surface.^[^ ^29^ ^]^ Similarly, the whitish deposits observed at the surface of altered enamel showed a powdery heterogeneous layer (Figure 3a–d). These deposits were observed at the surface of the green enamel and between the green enamel and the brown layer, corroborating that they were formed prior to the formation of the dark layer.

If this whitish layer is directly exposed to the environment, it can evolve to form lead sulfate (PbSO_4_), which has a lower Gibbs free energy of formation, i.e., is a compound more stable chemically that it is commonly formed in long term.^[^ ^32^ ^,^ ^33^ ^]^ However, when the lead carbonates are in contact with chlorine species, the formation of black Pb(IV) species and lead‐chlorinated compounds are favored following the reaction 1.^[^ ^36^ ^]^ The chlorinated species in the atmosphere could come from marine aerosols, road salts, as well as other natural sources. In England, high contents of chloride aerosols have been detected even at sites 100 km from the sea,^[^ ^42^ ^]^ which includes the locations where the windows were installed (Little Barrington, Mereworth, and Canterbury).

(1) $$2{\text{PbCO}}_{3}\cdot \text{Pb}{\left(\text{OH}\right)}_{2}+2\text{HOCl}⇌2{\text{PbO}}_{2}+{\text{PbCl}}_{2}+2H_{2}{\text{CO}}_{3}$$

The precipitation of PbO_2_ also agrees with the formation of these species in chlorinated water in lead water distribution systems,^[^ ^43^ ^]^ being the sequence of lead mineral‐phase development at pH 6.5–8:

(2) $$2{\text{PbCO}}_{3}\cdot \text{Pb}{\left(\text{OH}\right)}_{2}\Rightarrow {\text{PbCO}}_{3}\Rightarrow \beta -\text{Pb}O_{2}\Rightarrow \alpha -\text{Pb}O_{2}$$

## 4 Conclusions

The green enamel applied to some glass windows installed in the UK has spontaneously altered in a very fast way, forming a brownish layer that completely alters the artistic significance of the vegetal motifs on the windows. This green enamel is mainly composed of very high‐lead glass with cobalt and chromium as chromophores. The morphological observation of the surface showed the formation of two layers on it, one whitish and the other brownish on the top of the previous one, with a thickness of around 3 µm. FE‐SEM‐EDS and LIBS analyses confirmed that the brownish layer had higher lead content than the green enamel. The structural analysis verified the presence of scrutinyite (*α*‐PbO_2_) and plattnerite (*β*‐PbO_2_) in the brown layer, and hydrocerussite (2PbCO_3_·Pb(OH)_2_) and anglesite (PbSO_4_) in the whitish layer. The proposed alteration mechanism suggests that the green enamel was altered due to the environmental conditions, and the Pb^2+^ ion was released to the adsorbed water. This ion reacts with environmental gases such as CO_2_ and SO_2_ to form the carbonate and, after that, the sulfate. However, it seems that the hydrocerussite can also be transformed into lead oxide in environments with high chlorine contents, forming the brownish layer.

## Conflict of Interest

The authors declare no conflict of interest.

## Data Availability

The data that support the findings of this study are available from the corresponding author upon reasonable request.
